# Anti-Aliasing Attention U-net Model for Skin Lesion Segmentation

**DOI:** 10.3390/diagnostics13081460

**Published:** 2023-04-18

**Authors:** Phuong Thi Le, Bach-Tung Pham, Ching-Chun Chang, Yi-Chiung Hsu, Tzu-Chiang Tai, Yung-Hui Li, Jia-Ching Wang

**Affiliations:** 1 Department of Computer Science and Information Engineering, National Central University, Taoyuan 320, Taiwan; 2 Department of Biomedical Sciences and Engineering, National Central University, Taoyuan 320, Taiwan; 3 Department of Computer Science, University of Warwick, Coventry CV47AL, UK; 4 Department of Computer Science and Information Engineering, Providence University, Taichung 43301, Taiwan; 5 AI Research Center, Hon Hai Research Institute, New Taipei City 236, Taiwan

**Keywords:** computer-aided diagnosis, skin lesion segmentation, deep learning, light-weight model, medical internet of things

## Abstract

The need for a lightweight and reliable segmentation algorithm is critical in various biomedical image-prediction applications. However, the limited quantity of data presents a significant challenge for image segmentation. Additionally, low image quality negatively impacts the efficiency of segmentation, and previous deep learning models for image segmentation require large parameters with hundreds of millions of computations, resulting in high costs and processing times. In this study, we introduce a new lightweight segmentation model, the mobile anti-aliasing attention u-net model (MAAU), which features both encoder and decoder paths. The encoder incorporates an anti-aliasing layer and convolutional blocks to reduce the spatial resolution of input images while avoiding shift equivariance. The decoder uses an attention block and decoder module to capture prominent features in each channel. To address data-related problems, we implemented data augmentation methods such as flip, rotation, shear, translate, and color distortions, which enhanced segmentation efficiency in the international Skin Image Collaboration (ISIC) 2018 and PH2 datasets. Our experimental results demonstrated that our approach had fewer parameters, only 4.2 million, while it outperformed various state-of-the-art segmentation methods.

## 1. Introduction

Melanoma is one of the most prevalent types of cancer. It is the primary factor in most skin cancer deaths. In the United States in 2022, 99,780 cases were diagnosed as new melanoma, 57,180 in men and 42,600 in women. There were 7560 deaths by melanoma, with 5080 in men and 2570 in women. Compared with the report in 2020 by Worldwide, the number of patients diagnosed with melanoma was 324,635 people. Melanoma is considered as one of the deadliest types of skin cancer; early detection can extend survival by five years and increase the overall rate of survival. Thus, the demand for the diagnosis of melanoma in its early stages is increasing significantly. Furthermore, automated skin segmentation can be widely applied in many sensitive applications such as face tracking [1], face detection [2], and gesture recognition [3]. Other samples can be listed as content-based retrieval [4], robotics [5], virtual reality [6], face recognition [7], and human-computer interaction [8].

One solution for predicting melanoma is based on visual examination by dermatologists. However, this approach has become less popular due to its low accuracy, time-consuming nature, and reliance on human factors and trained experts. With the advancement of technology, machine learning and deep learning have emerged as promising techniques for predicting melanoma and other applications. The hope is that these methods can make the diagnosis of melanoma simpler, better, and more convenient. Deep learning has been applied to the prediction of skin segmentation, which supports melanoma diagnosis and significantly increases the efficiency of skin segmentation. The results also indicated that the effectiveness of previous approaches were dependent on equipment, color information of input datasets, personal factors, nonlinear illumination, and the availability of datasets. There are several factors that can affect skin lesion images, including the position of the lesion on the body, the type of equipment used to capture the image, and the presence of noise in the image [9,10]. Noise can manifest in various forms, such as ink spots, fuzzy borders, black hair, undesirable lighting, lesion artifacts, brown lesions, black hair with brown lesions, markers, white residues on the lesion, and hard scenes for the lesion. These factors can all pose significant challenges for accurate skin lesion segmentation. In order to improve segmentation performance, it is important to address these challenges and develop methods that can effectively handle the various types of noise and variability present in skin lesion images. Some factors affecting lesion skin images are as follows:

- Illumination: the skin color is affected by the level of illumination (shadows, nonwhite lights, and whether the image was captured indoors or outdoors).
- Camera equipment: the sensor quality of the camera directly impacts the color image.
- Ethnicity: people across different regions, as well as ethnic groups, have different skin colors.
- Individual characteristics: age and working environment also influence skin color.
- Other factors: background, makeup, or glasses also affect skin color.

**Figure 1:**
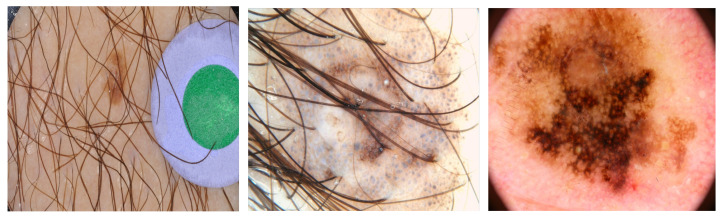
Some skin lesion images with noise.

Figure 1 illustrates some examples from skin lesion datasets with poor quality input images containing hair, noise from light, and resistance images. Moreover, the limited quantity of available images decreases the accuracy of skin segmentation. Prior studies have attempted to address these challenges by applying preprocessing steps to improve the quality of the input datasets. These preprocessing algorithms include resizing the input images, removing black hair, eliminating markers, and processing nondominant lesions and ink spots to enhance segmentation accuracy. However, these preprocessing steps can also increase the computational load on the system and prolong the processing time. In addition, if not carefully implemented, preprocessing algorithms can result in a loss of critical features from the input data.

**Figure 2:**
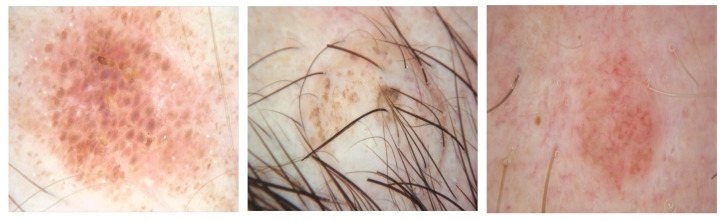
Uncertainty at the boundaries of the lesion.

Another difficulty of skin lesion segmentation is uncertainty at the boundaries of the lesion, as described in Figure 2.

Dermoscopy is a technique used to enhance the information obtained from skin lesions. By using a dermatoscope, information about the lesion can be seen more clearly, such as the presence of structures, colors, and patterns. However, even with dermoscopy, accurately delineating the boundary of the lesion remains a challenge, as well as determining the difference between healthy and lesion skin. These challenges highlight the need for further improvement in skin lesion segmentation techniques. Some prior studies tried to handle this problem. They used traditional segmentation methods, namely thresholding, colour-based segmentation algorithms, discontinuity-based segmentation, and region-based segmentation. Thresholding determines the threshold and divides the pixels into groups [11]. Color-based segmentation algorithms are based on color-discrimination segment images according to principle components and spherical coordinate transforms [12]. Discontinuity-based segmentation uses radial searching techniques or Laplacian or Gaussian zero crossing for segmentation [13]. Region-based segmentation [14] splits an image into small parts and segments by statistical region merging and multiregion growing. Recently, deep learning models have been published to perform skin segmentation. U-net is an example that is constructed with decoder and encoder paths [15]. Due to its simplicity and effectiveness, the u-net architecture was proposed in 2015 for biomedical image segmentation and has been considered state-of-the-art in this field ever since. Moreover, DeeplabV3 probes convolutional features at multiple scales corresponding to image-level features that encode the global context [16]. The feature pyramid network (FPN) is the most popular model for segmentation. It was designed with a pyramid of features to learn features at low and high resolutions [17]. E-net was designed from the ground up specifically for segmentation [18]. Mask R-CNN, which was introduced in 2017, has shown superior performance in instance segmentation tasks, where the goal is to detect and segment objects within an image [19].

The basic structure of all of these deep learning models consists of two main parts: an encoder and a decoder, also known as autoencoders. The encoder takes an input and maps it to a lower-dimensional representation, also known as a bottleneck. The decoder then takes this lower-dimensional representation and reconstructs the original input as closely as possible. The bottleneck is a crucial part of an autoencoder, as it forces the network to learn a compressed representation of the input data. This compressed representation can be used for various downstream tasks, such as clustering, classification, or anomaly detection. Autoencoders have several applications in various fields, such as the segmentation of images, language generation, translation, and sentiment analysis. In addition, autoencoders can be used for feature extraction, where the bottleneck is used to extract the most important features of the input data. This is particularly useful in domains such as computer vision and signal processing, where the input data can be highly dimensional. Although these models have primarily focused on improving the quality of datasets and segmentation efficiency for skin lesion segmentation, some of these studies have relied heavily on color cues, which can limit performance and lead to bias related to skin tones. Additionally, the availability of powerful GPUs has enabled more efficient deep learning processes. However, deep models with large numbers of parameters can be computationally intensive and impractical for use on personal computers. Therefore, there is a growing need for a lightweight model with fewer parameters that can be easily run on a personal computer. This problem is considered a subset of object segmentation and has been less addressed in previous studies.

In this study, we first considered overcoming the cons of the skin lesion dataset, which has a small available dataset and large noise from black hairs. Secondly, we designed a lightweight model to improve segmentation accuracy as well as ensure its real-time application. Overall, the highlighted aspect of this method is a lightweight model for skin segmentation that significantly enhances performance in segmentation, suppresses noise from input images, and overcomes the data-hungry problem. The proposed model with encoder and decoder paths can learn global context features and detailed local features in images to segment skin pixels in the input image. ISIC 2018, and PH2 are two available datasets that were used to train and evaluate the performance of the proposed method. Furthermore, we compared the performance of our proposed model with the performances of state-of-the-art methods. The primary contributions of this study can be summarized as follows:

- We proposed combining some augmentation techniques to robustly increase the quantity, as well as the quality, of the data. This suggestion overcame the noisy information in the input images, reduced the over-fitting problem, and increased the available images.
- We proposed a model with a multipath structure for robust skin segmentation that exploits both the context’s global and spatial features.
- The proposed model with an attention block can capture image highlights. It enhanced efficiency in training and testing and improved the accuracy of skin segmentation
- Results on the benchmark dataset showed the improvement of the proposed model, whereby our model with fewer parameters performed better than existing recognition methods.

## 2. Related Work

Traditional machine learning and deep learning methods are the two main approaches to skin lesion segmentation. For example, the authors used an ant colony as the traditional machine learning method to extract three lesion features: texture, color, and geometrical properties. They then used the K-nearest neighbour (KNN) technique to segment skin lesions [20]. Jaisakthi et al. [21] introduced combining the grab–cut algorithm and KNN for skin lesion segmentation. Adjed et al. [22] proposed the use of wavelet transforms and curvelet transforms to extract features, and then used SVM to classify skin lesion. Arroyo et al. [23] presented fuzzy histogram thresholding for segmenting skin lesions. Do et al. [24] applied hierarchical segmentation for skin lesion segmentation. Alfed et al. [25] integrated the HOG and SVM methods for skin lesion segmentation. Zhou et al. [26] segmented skin lesions using a gradient flow-based algorithm. Although these traditional machine learning methods can segment skin lesions, these methods need help with large datasets.

In truth, the development of computers and available big data urged researchers to implement deep learning for segmentation. Deep learning methods have achieved better performances in skin lesion segmentation. U-net, which is built upon FCNs with encoder and decoder paths, was considered as the state-of-the-art model for image segmentation. the total number of parameters in the u-net mode is approximately 31.03 million. The success of u-net in image segmentation led to the development of several updated models that built upon its architecture and introduced further advancements. For instance, DenseNetUnet [27] was built with numerous dilated convolutions with different atrous rates to observe a wide field of vision and utilized an attention mechanism with a better performance than that of u-net. The cons of the DenseUnet model are its huge number of parameters (it has 251.84 million parameters). Another example, called u-net 3+, is a modification of u-net that utilizes comprehensive skip connections and deep supervision [28]. The full-scale skip connections combine low-level details with high-level semantics from feature maps in multiple sizes. ResUnet++ is an extension of the u-net architecture that incorporates residual connections in the encoder and the decoder paths [29]. DoubleU-Net is another variant of the u-net architecture that includes Atrous Spatial Pyramid Pooling (ASPP) to selectively highlight contextual features [30]. Deep Residual u-net is a variant of the u-net architecture that contains residual connections and deep supervision to improve segmentation accuracy [31]. Furthermore, mask-RCNN is also a recent state-of-the-art model for segmentation. A mask-RCNN architecture includes an extension of a faster R-CNN that combines an existing path for bounding box identification with an approach for estimating an object mask. Segnet uses pooling in upsampling path indices calculated in the maximum pooling stage of the matching downsampling path [32]. VGG-16 focuses on an increased depth with a small filter size that has 138 million parameters [33]. Laddernet has skip connections between each pair of adjacent upsampling and downsampling paths in each level and many pairings of downsampling and upsampling paths. Deeplab v3 is NMF with an L0-constraint on the H columns (NMF L0-H) [34]. The total number of parameters in Deep lab v3 is 43.9 million parameters. Inception V3 utilizes a small filter size and has better feature representation [35]. The AASC model also has empirical results for skin lesion segmentation [36]. Recently, a novel boundary-aware transformer (BAT) has also shown impressive results for skin segmentation based on the development of transformer-based systems and a boundary-wise level of knowledge [37]. When comparing the diagnostic accuracy of an experienced dermatologist, a clinician with minimal dermoscopy training, and a computer-aided diagnosis, it was found that the former two had lower sensitivity (69%) than the computer (92%) [38]. Overall, these deep learning methods perform better skin segmentation than traditional machine learning methods, but the model sizes are large with computational complexity. Thus, these deep learning models have slow training and inference times. Furthermore, these models require significant preprocessing to handle varying input sizes. These deep learning models are prone to overfitting when the training dataset is small. In this study, we propose a lightweight model with high performance in skin lesion segmentation compared to existing techniques.

## 3. Proposed Method

**Figure 3:**
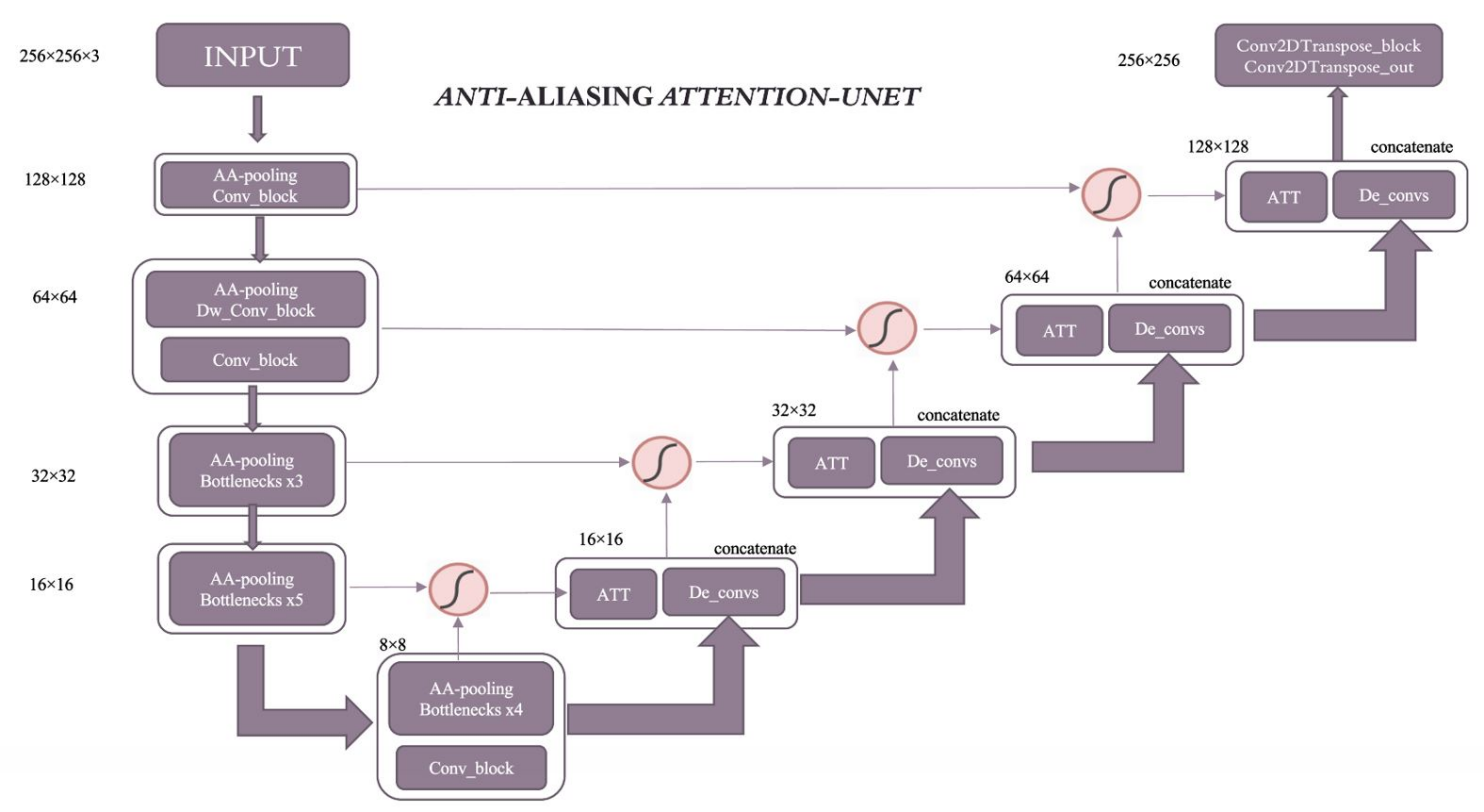
Anti-aliasing Attention U-net Model.

An overview of the proposed model is shown in Figure 3.

**Figure 4:**
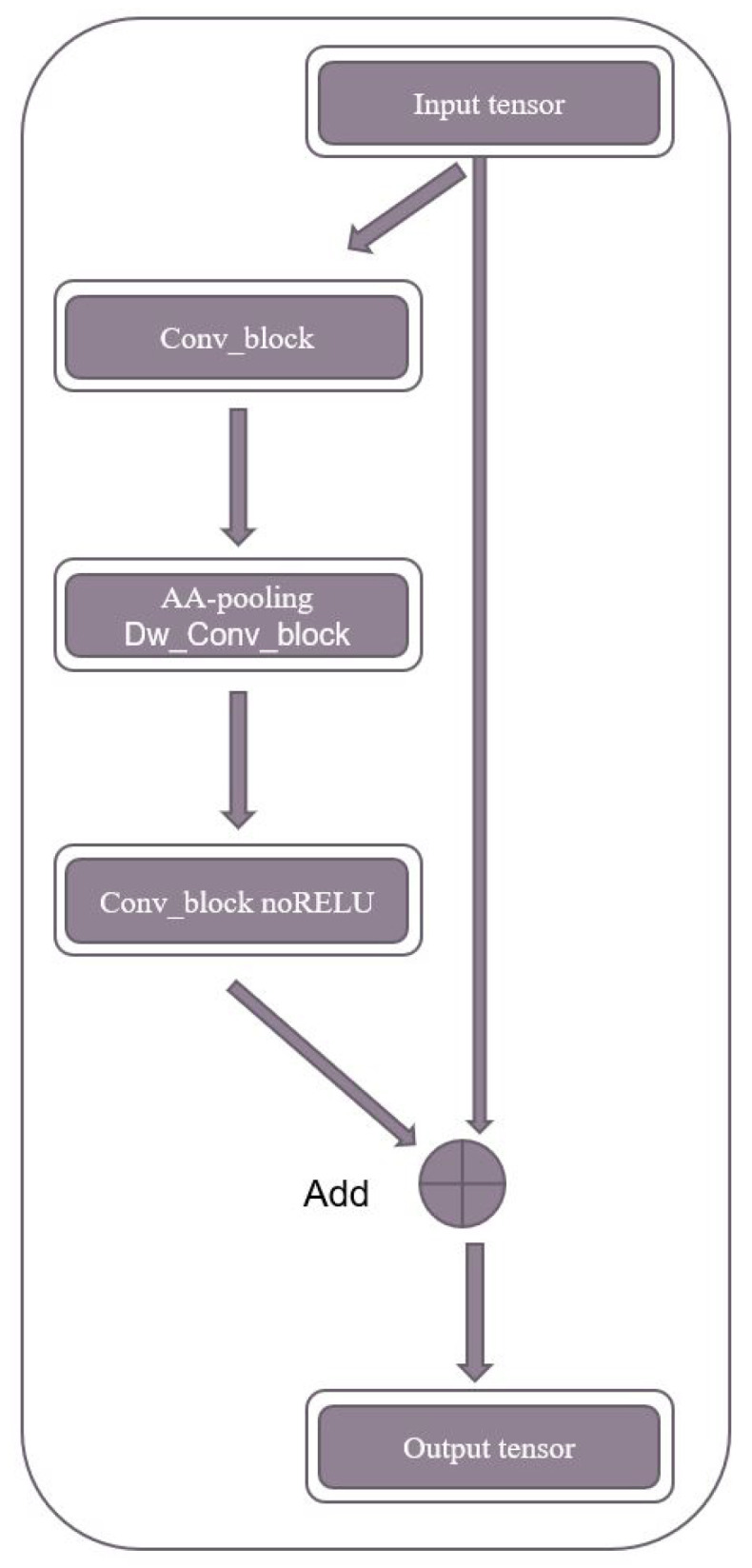
The bottleneck architecture.

The MAAU model is a lightweight model designed for skin lesion segmentation. It consists of two main parts: an encoder path and a decoder path. The encoder path is responsible for extracting meaningful features from the input image, while the decoder path is responsible for reconstructing the original image by decoding the encoded feature maps from the encoder path to produce a segmentation mask. The encoder path begins with an anti-aliasing layer (AA-pooling), which reduces the spatial resolution of the input image while avoiding shift equivariance [39]. Transparently, input images are reduced four times in the encoder, from 256 × 256 to 16 × 16. After the AA-pooling layer, the output is passed through a series of convolution blocks (Conv_blocks) and bottleneck blocks. The Conv_blocks consist of 2D convolution, followed by batch normalization and a rectified linear unit (ReLu). The batch normalization in the Conv_blocks helps to regularize the model and reduce the internal covariant shift, while the ReLu adds nonlinearity. The bottleneck block in the encoder is a unique feature that combines Conv_blocks, AA-pooling, depthwise convolution (Dw_Conv_block), and Conv_blocks without ReLu to summarize input tensor features and produce the final components. Figure 4 provides a detailed illustration of the bottleneck block’s components and their interactions. By leveraging these techniques, the MAAU model achieves accurate skin lesion segmentation while being lightweight and efficient.

**Figure 5:**
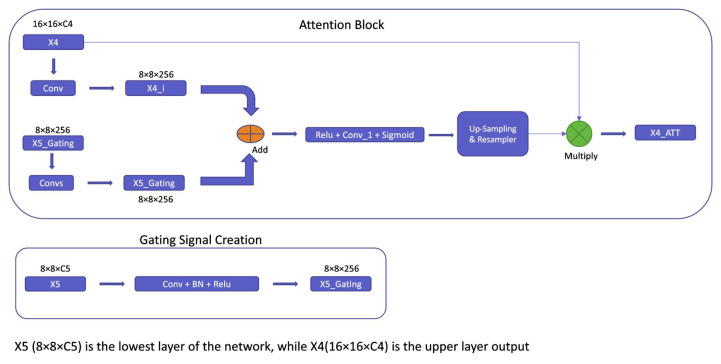
The attention block.

**Figure 6:**
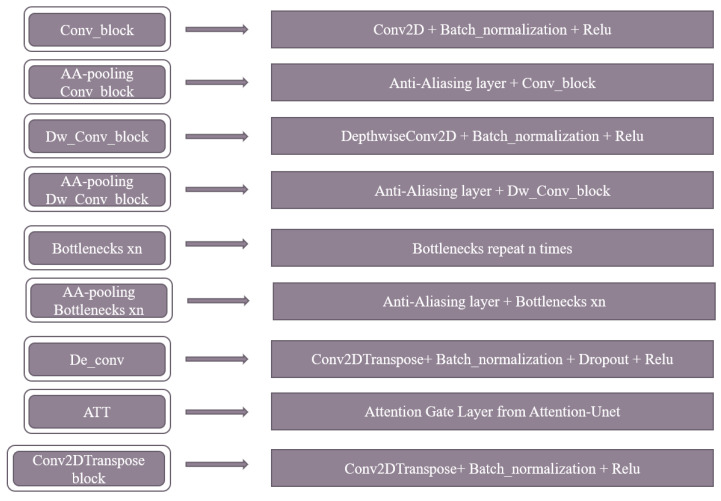
The model’s details.

Another important aspect of the MAAU model is its decoder, which is responsible for upsampling the encoded feature maps from the encoder to produce a segmentation mask. The decoder consists of two main components: the attention block (ATT) and the decoder module (De_convs). The attention block is designed to capture important features in each channel of the input feature map, as shown in Figure 5. The value $X_{i}$ (8 × 8 × $C_{i}$) is the lowest layer of the network, and $X_{j}$ (16 × 16 × $C_{j}$) is the upper layer output. The values $X_{i}$ and $X_{j}$ are the two input values of the AAT, and $C_{i}$ and $C_{j}$ represent the channels of $X_{i}$ and $X_{j}$, respectively. The attention block uses an attention gate layer from attention u-net, which considers every channel and its neighbor to capture local cross-channel interactions [40]. This enables the attention block to effectively capture the most relevant information in each channel of the feature map. The output of the attention block is then passed through a series of decoder modules (De_convs), which consist of Conv2DTranspose, Batch_normalization, Dropout, and ReLu layers. These layers increase the size of the feature map and add additional features to help produce a more accurate segmentation mask. Finally, a convolution2D transpose block (Conv2DTranspose_block) is used to further increase the size of the feature map, and a sigmoid activation function is applied to produce the final segmentation mask. It is worth noting that the MAAU model also includes auxiliary connections between each pair of encoder and decoder layers. These connections allow for additional information to be passed between layers, which improves the overall performance of the model. The detailed architecture of the MAAU model is shown in Figure 6. Overall, the MAAU model’s lightweight design, coupled with its attention-based decoder and auxiliary connections, make it a promising approach for skin lesion segmentation. By combining anti-aliasing pooling, attention blocks, and an encoder–decoder architecture, the model can achieve high accuracy while minimizing the number of parameters required.

## 4. Experiments

### 4.1. Dataset

#### 4.1.1. Dataset Modalities

First, we trained and tested the proposed method on dermoscopic skin images from the ISIC 2018 [41] dataset. To evaluate the proposed model’s performance, we divided 2594 images into 5 folds for cross-validation. This task was handled using sklearn.model_selection.KFold. For each unique group, the following was performed:

- One fold was used as the testing dataset.
- The remaining folds were used as the training dataset.
- The model was fitted on the training dataset and evaluated on the testing dataset.
- An average of five tests was used to obtain the final result.

Second, we conducted training of our proposed model on the ISIC 2017 dataset and evaluated its performance on 200 dermoscopic images from the PH2 dataset, following the methodology of a previous study [42].

#### 4.1.2. The Preprocessing Dataset

In this study, to improve the performance of our skin segmentation model, we applied several augmentation techniques to the input images. We started by resizing the images to 256 × 256. Then, we applied various spatial functions, such as vertical/horizontal flip, translate, shear, and rotation, to create more diverse images, which helped the model to generalize more effectively. Additionally, we used color distortions, such as brightness, contrast, saturation, and hue, to enhance the quality of the dataset further. To reduce noise in the input images, we applied adaptive histogram equalization and Gaussian blur. Overall, these augmentation techniques increased the diversity and quality of the dataset, which led to better model performance.

### 4.2. Experimental Setups

The implementation was performed in python, and the experimental results were run on CPU I7-9700 with ONNX. The inference speed was 67 FPS. We used Tensorflow as the framework for our implementation. For the optimizers, we set the model with the Adam optimizer at the default setting using the initial learning rate of 0.001, beta 1 = 0.9, beta 2 = 0.999, and epsilon = $1\times 10^{-7}$. We trained the model in 200 epochs with the Dice loss function, a popular loss function in image segmentation.

To deal with the over-fitting problem, “ReduceLROnPlateau” and “EarlyStopping” were our choices. The first method sets a learning rate schedule by reducing the learning rate by a factor of “0.2” once if there is no improvement in the “ model’s loss” for 20 epochs; the second method terminates the training procedure if there is no improvement in 50 epochs.

### 4.3. The Evaluation Protocol

The experimental results were evaluated by metrics such as the Dice coefficient, Jaccard cofficient, precision, recall, accuracy, and the F1-score [43].

(1) $$Dice=\frac{2*(X\cap Y)}{X+Y},$$

where *X* and *Y* are two sets. The operator ∩ is described as the point where the two sets intersect. To enhance the similarity calculation between two sets, we used the Jaccard index, which is known as the Jaccard similarity coefficient.

(2) $$Jacc=\frac{|X\cap Y|}{|X|+|Y|-|X\cap Y|}.$$

Any segmentation error, whether over-segmentation or under-segmentation, reduced the scores of the Jaccard index. Precision, recall, accuracy, and the F1-score were used as criteria for pixel-level evaluation. The formulation of precision, recall, accuracy, and the F1-score are described below:

(3) $$Precision=\frac{TP}{TP+FP}$$

(4) $$Recall=\frac{TP}{FN+TP}$$

(5) $$Accuracy=\frac{TP+TN}{TP+TN+FP+FN}$$

(6) $$F1-score=\frac{2*Precision*Recall}{Precision+Recall}$$

where *TP* is true positive, *FP* is false positive, and *FN* is false negative.

## 5. Results

### 5.1. Quantitive Results

**Figure 7:**
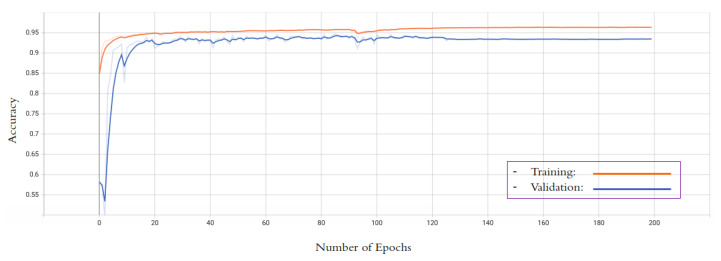
The training and testing processes evaluated by the accuracy metric.

**Figure 8:**
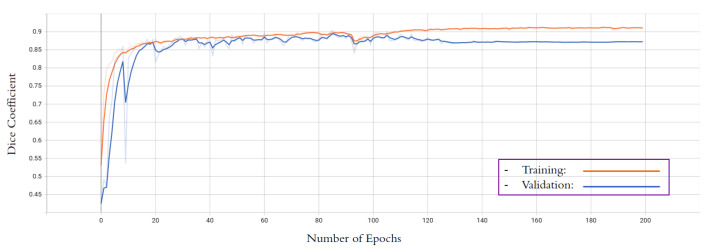
The training and testing processes evaluated by the Dice coefficient.

The performance of the model was evaluated using images from the ISIC 2018 dataset, and the results were assessed through a five-fold cross-validation process. Figure 7 shows the accuracy metric of the model for the training and testing data. It can be observed that the training process (orange line) and the testing process (blue line) remained stable over 200 epochs, indicating that the model converged. Moreover, the experimental results were shown to be high, indicating that the model performed well. Figure 8 illustrates the training and validation processes of the model over 200 epochs, evaluated by the Dice coefficient. The training process results were generally higher than the evaluation process results, but both remained stable over the 200 epochs. This suggested that the model is capable of producing consistent and reliable results.

Table 1 shows the evaluation metrics and corresponding scores for the proposed mobile anti-aliasing attention u-net (MAAU) model on the ISIC 2018 dataset. The evaluation metrics used in this study were the Dice coefficient, the Jaccard index, precision, recall, accuracy, and the F1-score. The Dice coefficient score of the MAAU model on ISIC 2018 was 0.881, which indicated the extent to which the predicted segmentation aligned with the ground truth segmentation through overlap analysis. The Jaccard index score of the MAAU model on ISIC 2018 was 0.809, which measured the resemblance between the predicted and ground truth segmentation. The precision score of the MAAU model on ISIC 2018 was 0.902, which calculated the ratio of true positive pixels (accurately segmented skin lesion pixels) to the total number of pixels predicted to be positive. The MAAU model achieved a recall score of 0.909 on the ISIC 2018 dataset, indicating the proportion of true positives to the total number of positive pixels in the ground truth. The accuracy score of the MAAU model on the ISIC 2018 dataset was 0.955, which measured the proportion of correctly segmented skin lesion pixels in proportion to the total number of pixels or skin lesion pixels. Finally, the F1-score of the MAAU model on the ISIC 2018 dataset was 0.906, which provided a measure of the balance between the precision and recall metrics. Overall, the proposed MAAU model achieved a high performance on the ISIC 2018 dataset, as evidenced by the high scores across all six evaluation metrics.

Table 2 shows a performance comparison between our proposed mobile anti-aliasing attention u-net (MAAU) model and several other methods on the ISIC 2018 dataset. The table presents the Dice and Jaccard metrics for each method. As we can see from the table, the best performance for the Dice coefficient was achieved by DoubleU-net with a score of 0.896. For the Jaccard coefficient, the best performance was achieved by TransUNET with a score of 0.822. Our proposed MAAU method achieved a Dice score of 0.881 and a Jaccard score of 0.809, outperforming u-net, Unet++, MultiResUnet, and DeeplabV3. Despite achieving slightly lower scores in both Dice and Jaccard coefficients compared to DoubleU-net and TransUNET, with a difference of 0.015 and 0.013, respectively. Our proposal offers the advantage of a simpler architecture that requires shorter training times and lower computational requirements. Our model design has fewer parameters, only 4.2 million against 29.3 million (DoubleU−net) and 105.3 million (TransUNET). In summary, the outcomes showcased the efficacy of the method we proposed for segmenting skin lesions.

Table 3 shows the performance of our proposed method and previous studies on the PH2 dataset dataset, as evaluated by the Jaccard index (Jacc). We implemented this according to the study in [42]. Our proposed method achieved the highest score of 0.845, outperforming all previous methods, including CDNN, U-NET, FCN, ResNet, VGG16, and Res-Unet. This indicated that our proposed method has the potential to be an effective solution for skin lesion segmentation tasks.

Table 4 lists several deep learning models along with the number of parameters in millions for each model. The models include u-net, Unet++, DoubleU-net, MultiResUnet, TransUNET, DeeplabV3, and our proposal. The number of parameters in each model ranged from 4.2 million, in our proposed method, to 105.3 million, in TransUNET. Overall, our proposed method had the lowest number of parameters among all the models listed. This suggested that the proposed method might have a simpler architecture than the other models, which could lead to faster training times, lower computational requirements, and be implemented in practical applications with limited computational resources.

### 5.2. Qualitative Results

**Figure 9:**
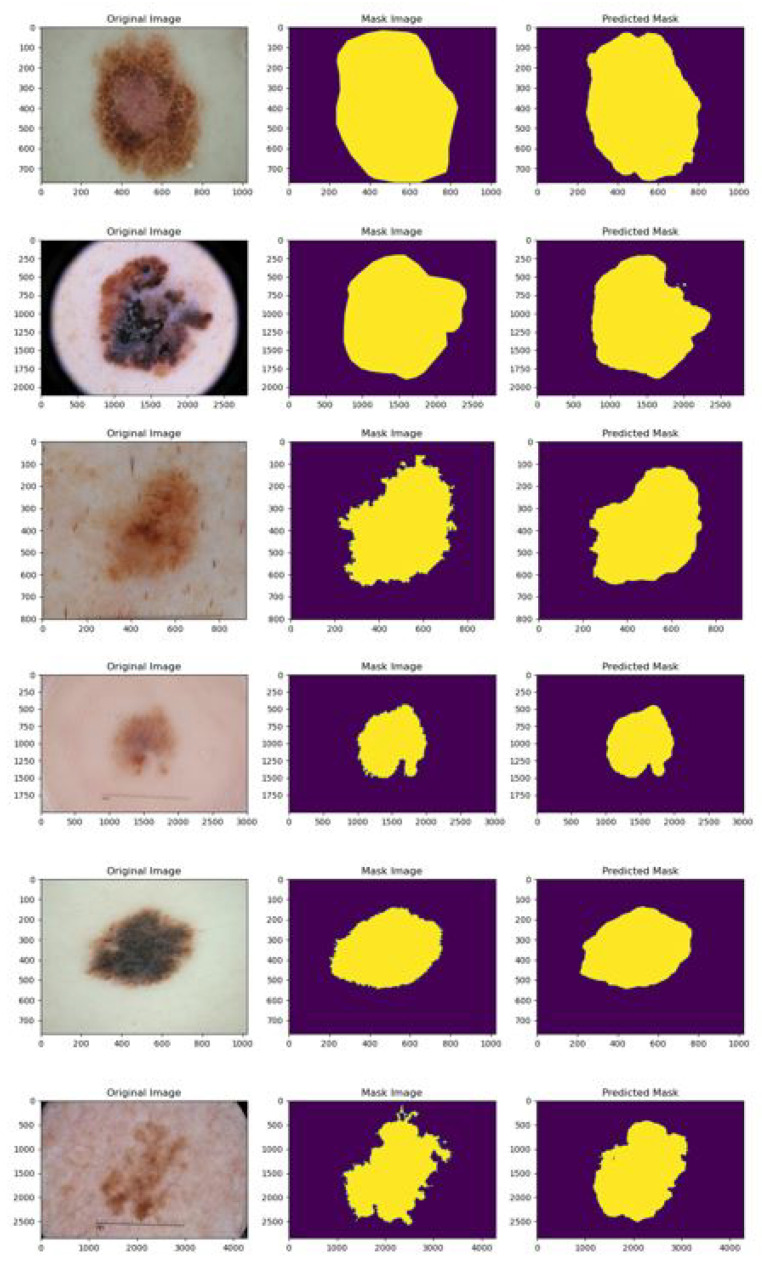
Qualitative results of MAAU on the ISIC 2018 dataset.

The visualization of the experimental results is depicted in Figure 9. Prior to being fed into the MAAU model, the original images were preprocessed. The mask images, which were used for evaluation, are available in the ISIC 2018 and PH2 datasets. The predicted masks shown in the figure were the segmented result images of the MAAU model. It was evident that the predicted masks closely resembled the mask images. This indicated that the proposed method effectively addressed the challenges related to light, color information, resistance images, and uncertainty at the boundaries in the data construction process. The preprocessing step significantly enhanced the data representation and the efficiency of the model. The obtained results demonstrated that the proposed MAAU model performed well in skin lesion segmentation.

## 6. Conclusions and Future Directions

In this study, we proposed a mobile anti-aliasing attention u-net model for skin lesion segmentation. The MAAU has some main components: lightweight mobile blocks, attention u-net structure, and anti-aliasing as a pooling layer. The visualization in the experimental results showed that the mask images and predicted masks were quite similar. The visualization demonstrated transparently that the proposed method overcame the drawbacks in the data to segment skin lesions accurately. The performance of the MAAU was better compared to the baselines of u-net, Unet++, DeeplabV3, and MuiltiResUnet. While the result of the MAAU model on the ISIC 2018 dataset was lower than DoubleU-net and TransUNET by approximately 0.01 and 0.02 in terms of the Dice coefficient and Jaccard index, respectively. This study’s distribution employed a lightweight model of only 4.2 million parameters, which is less than that of previous models. It is flexible, enabling the proposed model to be implemented in an individual computer without a GPU.

In the future, we plan to test the model’s efficiency on multiple datasets. Moreover, we will focus on simplifying the architecture while retaining its production ability. The system could self-organize with a nonfixed structure to respond efficiently to dynamic changes in datasets. This method is useful and robust for increasing the reliability and robustness of the service-continuity network. It could be applied to the accurate diagnosis of skin melanoma (SM) in clinical digital-dermoscopy images (DNNs), in particular, and relevant healthcare imaging applications in general. Furthermore, this approach could deal with the computational cost and resource constraints in service-oriented networks (SONs).

## Figures and Tables

**Table 1:** Experimental results on the ISIC 2018 dataset.

Metrics	Dice	Jacc	Precision	Recall	F1-Score	Accuracy
ISIC 2018	0.881	0.809	0.909	0.893	0.900	0.955

**Table 2:** Performance comparison with the previous studies on the ISIC 2018 dataset.

Method	Dice	Jaccard
U-net [40]	-	0.764
Unet++ [40]	0.879	0.805
DoubleU-net [40]	0.896	0.821
MultiResUnet [40]	-	0.803
TransUNET [40]	0.894	0.822
DeeplabV3 [40]	0.884	0.806
Our proposal	0.881	0.809

**Table 3:** Performance comparison with the previous studies using the PH2 dataset.

Method	Jaccard
U_NET [42]	0.762
FCN [42]	0.760
ResNet [42]	0.758
VGG16 [42]	0.754
Res-Unet [42]	0.772
Our proposal	0.845

**Table 4:** Parameter comparison with the previous studies.

Model	Parameters (Million)
U-net	7.7
Unet++	9.0
DoubleU-net	29.3
MultiResUnet	7.3
TransUNET	105.3
DeeplabV3	81.3
Our proposal	4.2

## Data Availability

The data presented in this study are openly available at https://challenge.isic-archive.com (accessed on 8 January 2018) [41], and at https://www.fc.up.pt/addi/ph2%20database.html (accessed on 3 July 2013) [42].
